# 12.3 SYSTEM XC- AS A NOVEL MODULATOR OF CORTICOSTRIATAL NEUROTRANSMISSION

**DOI:** 10.1093/schbul/sby014.046

**Published:** 2018-04-01

**Authors:** Eduard Bentea, Cynthia Moore, Agnès Villers, Madeline J Churchill, Rebecca L Hood, Lauren Deneyer, Lise Verbruggen, Giulia Albertini, Hideyo Sato, Laurence Ris, Charles K Meshul, Ann Massie

**Affiliations:** 1 Vrije Universiteit Brussel; 2 VA Medical Center, Portland; 3 Université de Mons; 4 Oregon Health & Science University; 5 Niigata University; 6 Oregon Health & Science University, VA Medical Center

## Abstract

**Background:**

System xc- is a plasma membrane amino acid antiporter, of mainly glial origin, that couples the import of cystine with the export of glutamate. System xc- (specific subunit xCT) contributes substantially to ambient extracellular glutamate levels in various regions of the brain, including the striatum and hippocampus. Despite the fact that system xc- is highly expressed in the brain and is a proposed therapeutic target for various neurological disorders, its function under physiological conditions in the central nervous system remains poorly understood. By acting as a source of glial extrasynaptic glutamate, system xc- might modulate synaptic transmission as a mechanism of neuro-glial communication. Previous electrophysiological findings indicate that system xc- delivered glutamate can inhibit excitatory synaptic neurotransmission in the corticoaccumbens pathway and at hippocampal CA3-CA1 synapses. To gain further insight into the proposed function of system xc- as modulator of synaptic transmission, we here focus on corticostriatal synapses.

**Methods:**

Single section electron microscopy was carried out on VGLUT1-pre-embed and glutamate immunogold post-embed labeled slices of the dorsolateral striatum of xCT+/+ and xCT-/- mice. Various parameters related to the pre- and post-synaptic compartments were integrated on the obtained electron micrographs, including glutamate immunogold density in the presynaptic terminal and spine, area of the terminal and spine, measures of the postsynaptic density (PSD) (length, area, thickness, and maximum thickness), percentage of PSDs showing perforations, and width of the synaptic cleft. Electrophysiological measures of corticostriatal transmission were obtained by recording the amplitude of field excitatory postsynaptic potentials (fEPSPs) after stimulation of corticostriatal fibers. Finally, grooming behavior was compared between xCT-/- and xCT+/+ littermates.

**Results:**

Genetic deletion of xCT led to depletion of glutamate immunogold labeling from corticostriatal terminals and their corresponding dendritic spines. Absence of xCT did not, however, affect the morphology of corticostriatal synapses, as evaluated by the area of the terminals and spines, size of the PSD, and width of the synaptic cleft. Similarly, no changes could be observed in the density of VGLUT1-positive synapses, indicating normal cortical innervation and spine density. Electrophysiological recordings revealed decreased amplitude of fEPSPs in xCT-/- mice after stimulation of corticostriatal fibers. Preliminary investigations revealed that this reduced response can be rescued by restoring physiological levels of glutamate to xCT-/- slices. Changes in corticostriatal transmission were not reflected in aberrant grooming behavior in xCT-/- mice; we could not observe any difference in the total grooming duration, the number of grooming bouts, the average bout duration or the latency to onset to grooming between xCT-/- and xCT+/+ mice.

**Discussion:**

Contrary to available evidence at hippocampal and corticoaccumbens pathways, our findings indicate a positive effect of system xc- on basal synaptic transmission at corticostriatal synapses. The decreased response we observed after stimulation of corticostriatal fibers in xCT-/- mice was accompanied by depletion of glutamate immunogold labeling from corticostriatal terminals, suggesting a possible defect in presynaptic glutamate handling. Given the strong decrease (70%) in extracellular glutamate levels previously reported in this strain of mice, we hypothesize that the decreased presynaptic glutamate labeling in xCT-/- mice is related to a loss of extracellular glutamate needed to supply terminals for proper excitatory transmission. This hypothesis is supported by our preliminary results showing increased responses in xCT-/- slices after restoring physiological levels of glutamate. Together, our findings shed new light on the role of system xc- in controlling synaptic transmission, and suggest that it may play an important role in supplying presynaptic terminals with glutamate as an alternative mechanism to the glutamate-glutamine cycle. As a novel modulator of corticostriatal transmission, system xc- may be of interest as a possible therapeutic target for disorders with a corticostriatal component, such as schizophrenia or obsessive-compulsive disorder.

